# Predictive Physiological Modeling of Percutaneous Coronary Intervention – Is Virtual Treatment Planning the Future?

**DOI:** 10.3389/fphys.2018.01107

**Published:** 2018-08-13

**Authors:** Rebecca C. Gosling, Paul D. Morris, Patricia V. Lawford, D. Rodney Hose, Julian P. Gunn

**Affiliations:** ^1^Department of Infection, Immunity and Cardiovascular Disease, University of Sheffield, Sheffield, United Kingdom; ^2^Department of Cardiology, Sheffield Teaching Hospitals NHS Foundation Trust, Northern General Hospital, Sheffield, United Kingdom; ^3^INSIGNEO Institute for in Silico Medicine, Sheffield, United Kingdom; ^4^Department of Circulation and Medical Imaging, Norwegian University of Science and Technology, Trondheim, Norway

**Keywords:** computational modeling, coronary artery disease, percutaneous coronary intervention, coronary physiology, predictive modeling

## Abstract

Computational modeling has been used routinely in the pre-clinical development of medical devices such as coronary artery stents. The ability to simulate and predict physiological and structural parameters such as flow disturbance, wall shear-stress, and mechanical strain patterns is beneficial to stent manufacturers. These methods are now emerging as useful clinical tools, used by physicians in the assessment and management of patients. Computational models, which can predict the physiological response to intervention, offer clinicians the ability to evaluate a number of different treatment strategies *in silico* prior to treating the patient in the cardiac catheter laboratory. For the first time clinicians can perform a patient-specific assessment prior to making treatment decisions. This could be advantageous in patients with complex disease patterns where the optimal treatment strategy is not clear. This article reviews the key advances and the potential barriers to clinical adoption and translation of these virtual treatment planning models.

## Introduction

Computational modeling techniques are employed routinely in the pre-clinical development of medical devices. In this context, modeling allows rapid prototyping, which is both time-and cost-effective. Yet, few models have entered the clinical domain as either diagnostic or predictive treatment-planning tools. *In silico* models of the cardiovascular system are amongst the most advanced. The recent adoption of CT-FFR into the major clinical guidelines represents a major breakthrough (Koo et al., 2011; NICE, 2016). However, this has not been universally adopted by healthcare professionals (Schoenhagen and Desai, 2015; Davies and Cook, 2017). The emergence of such tools has been hampered by difficulties with validation, regulatory approval, and lengthy processing times (Morris et al., 2015). The nature of predictive computational modeling is appropriate for virtual treatment planning, especially in the context of structural cardiovascular intervention. Clinicians frequently make treatment decisions based upon data pooled from randomized controlled trials, which can be problematic. First, these population-level data are extrapolated and applied to individuals. Second, randomized trials frequently recruit younger, otherwise well patients, and therefore under-represent the “average” patient who is typically older, with multiple comorbidities. Medicine requires an approach more tailored to the individual patient, based upon patient-specific characteristics. As one example, existing computer models created for the purpose of device design can be adapted to permit virtual treatment planning with the addition of patient specific geometries and personalized parameterization. In CAD, there is an opportunity to improve treatment planning. PCI treatment planning is often subjective. Decisions regarding the number, size, and position of stent(s) required to treat a coronary artery lesion(s) are made by the operator based upon a visual interpretation of the angiogram, a method which is frequently flawed due to the difficulty inferring the physiological impact of atherosclerotic lesions, and indeed their likely response to treatment, from 2D anatomical imaging (White et al., 1984). The development of virtual stenting tools may allow predictive treatment planning. This is emerging as an area of increasing clinical interest. This article reviews the rationale and developing methodology behind virtual PCI tools, the current state of the art, and what barriers need to be overcome before this patient-specific approach can be fully translated and incorporated into routine medical practice.

### What Is Computational Modeling, and How Can It Be Applied to CAD?

Computational models simulate the behavior of systems combining mathematics, physics, and computer science. Computational modeling techniques have been used for decades in engineering applications, and some of these techniques are particularly applicable in the study of CAD, namely CFD and FEM. CFD is a numerical technique that predicts and analyses mechanical responses of fluids to external (and other) forces allowing the quantification of physiological parameters such as blood flow velocity and pressure. Furthermore, the use of FEM can provide full and detailed quantitative stress and strain analysis, which can be applied to the vessel wall (Morris et al., 2016). These models can be manipulated to simulate states of disease and are especially relevant in the study of CAD where the clinical importance of physiology has been recognized in recent years.

A number of invasively measured physiological parameters have been developed that can be used to guide treatment decisions (Pijls et al., 1993; Meuwissen et al., 2002; Sen et al., 2012; van de Hoef et al., 2012, 2016). These can describe the effect of a coronary lesion on blood flow, pressure, and the relationship between the two. FFR, the pressure drop measured across a lesion at maximal hyperaemia, is now considered the gold standard measure to determine coronary artery lesion significance (NICE, 2016). Using FFR to guide PCI is associated with improved clinical outcomes (De Bruyne et al., 2012). Furthermore, other physiological indices, that cannot be measured invasively, such as WSS are increasingly being recognized as factors that influence outcomes such as the rate of development of ISR (Tahir et al., 2011). With the application of CFD and FEM modeling, it is possible to predict the effect of stenting on these parameters, which can be useful in both stent design and patient-specific treatment planning.

### Modeling Coronary Artery Stents

Computational methods are routinely used in designing stents and in predicting their performance and fatigue. They can also be used to model the effect of the stent upon blood flow in a diseased artery at the strut level, where disturbed flow can be a causative factor in the development of in-stent thrombosis, restenosis, and neo-atherosclerosis. Modeling is particularly applicable to study these phenomena, which are beyond the level of resolution of clinical measurements of flow (Van der Heiden et al., 2013). Neointimal thickening after stenting is related to altered local fluid dynamics (low and oscillating WSS provoked by the presence of the stent within the coronary artery) (Timmins et al., 2011). A number of models of ISR have been developed, and a relationship between stent design parameters such as strut thickness and the shape and depth of strut deployment within the vessel wall on the severity of ISR is well established (Tahir et al., 2011). Such models can be used to assist with stent design and to predict local hemodynamic effects of stenting and have been reviewed elsewhere (Martin and Boyle, 2011). The advancement of these models has received significant support from industry, and the technology is well developed. Applying the same technology to patient-specific geometries allows virtual treatment planning and is a growing area of interest. The two main thrusts of being in bifurcation modeling and modeling FFR.

## Treatment Planning in Bifurcation Disease

Percutaneous coronary intervention of bifurcation lesions is beset by poorer results than non-bifurcation lesions (Lassen et al., 2014; Sawaya et al., 2016). Multiple technical strategies have been proposed, and the optimal strategies are still an area of debate (Lassen et al., 2016; Sawaya et al., 2016). Computational simulations offer key information on the biomechanical effects of stenting. Such simulations enable virtual testing of various strategies and can assist in evaluating outcomes.

### What Is Special About Bifurcations?

Bifurcations are more prone to atherosclerosis due to the development of adverse flow patterns leading to regions of low WSS developing opposite the side branch and down the lateral wall of the branch itself. PCI to the main vessel is complicated by the risk of side branch occlusion due to plaque shift. Often multiple stent strategies are employed, which increases the risk of vascular damage and ISR. A number of studies have successfully utilized computational models to examine the impact of different stent designs and techniques on local hemodynamic factors (Williams et al., 1985; Gastaldi et al., 2010; Morlacchi et al., 2011, 2014; Mortier et al., 2015). Such models have advanced in terms of sophistication over the past 5–10 years. These studies have assisted with advancements in the modeling of stent insertion and have provided valuable insights into the relationships between stent design, WSS, and ISR. However, only with patient-specific models can accurate patient-specific treatment planning be achieved.

### Anatomical Representation of Bifurcations

The first challenge in modeling bifurcations using patient-specific models is in the segmentation (reconstruction) of the patient anatomy. This is complex due to the necessity for precision of the cross-sectional area, branch diameter, and branching angle. The more realistic the models, the more insightful the investigations. Because of the difficulty faced reconstructing a complex bifurcation anatomy from invasive coronary angiography alone, many of these models utilize information from invasive imaging such as IVUS and OCT. Various methods are complementary and many hybrid combinations have been tested (Morlacchi et al., 2011; Mortier et al., 2015; Wang et al., 2015; Chiastra et al., 2016). The addition of numerical simulations of mechanical stresses and fluid flow in patient-derived geometries can contribute to translational experimentation. Modeling can also compare the expected results with different stent designs and strategies.

### Complementary Imaging to Assist Bifurcation Modeling

**FIGURE 1:**
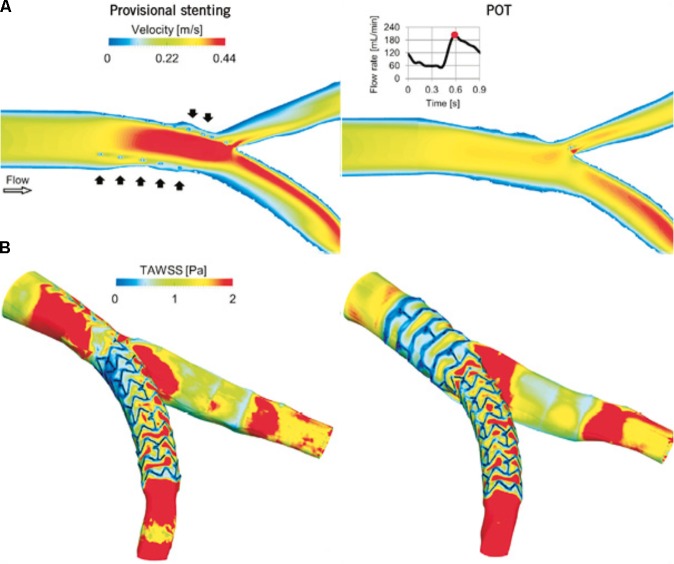
Computational replication of bifurcation stenting. Mortier et al. (2015) compared flow velocity patterns and WSS following virtual provisional stenting (left) and proximal optimization technique (right). **(A)** Velocity contour maps on an internal plane of the bifurcation at peak flow rate. In the provisional stenting, where the malapposed struts are clearly visible (black arrows), higher velocities are present as the stent behaves as a cylindrical hurdle in the flow stream. Indeed, in the proximal optimization technique (POT), struts are well opposed to the wall and do not obstruct the blood stream. **(B)** Time-averaged WSS (TAWSS) contour maps. Low WSS are generated next to the stent struts. A wide area with low WSS is present in the bifurcation region in the provisional stenting model and in the proximal part of the stent in the POT model. Reprinted from Mortier et al. (2015). Copyright (2015), with permission from Europa Digital & Publishing.

Mortier et al. (2015) used CTCA and IVUS pullbacks to create a patient-specific virtual 3-D model. Using FEM, they generated stent and balloon models, accurate in terms of geometry and mechanical behavior, allowing them to perform and evaluate different stenting strategies. Transient CFD analysis was performed to produce velocity patterns and examine WSS along the arterial wall after stent deployment. Stents could be repositioned to investigate the impact upon WSS distributions, the optimal position being associated with minimal area of low WSS (Mortier et al., 2015) (**Figure 1**). Similar work has been achieved combining CTCA with OCT. Chiastra et al. (2016) reconstructed patient-specific models of coronary bifurcations from CT-OCT. They demonstrated good qualitative geometrical correlation between post-operative lumen area after virtual stent expansion and that from hybrid CT-OCT. They demonstrated the ability to determine the best stent position to minimize the percentage of mal-opposed struts (Chiastra et al., 2016). Wang et al. (2015) created a patient-specific bifurcation model from angiographic images alone. As well as using FEM analysis to identify areas of low WSS associated with four different stenting approaches, they also used 3-D printing to create an *in vitro* model. Using microfabrication, microfluidic chips implanted with real stents were used to mimic PCI with real time visualization. The results from their 3-D models were highly consistent with simulated results. This model has the advantage of allowing the testing of positioning effects of real stents experimentally. This approach may be more translational as it is subject to the same difficulties of such precise stent positioning faced *in viv*o.

### Limitations of Bifurcation Modeling

One problem is that the above methods considerably add to the complexity of a PCI procedure. Most bifurcations can be treated reasonably well with 2-D angiographic guidance. However, in select patients with complex anatomy, these techniques could allow detailed treatment planning to occur prior to PCI. Demonstrating clinical benefit would also be challenging as the results of conventional angiographically guided PCI are generally good, masking particular benefit to be derived by a minority of patients with complex 3-D anatomy. Moreover, replicating exact stent positioning recommended by the model in the catheterization laboratory would be challenging and this could be a major barrier to clinical translation. At present, significant processing time is required as these models incorporate FEM technologies. This limits the option of “live” modeling in the cardiac catheter laboratory. These models may be able to assist in demonstrating the benefit of one strategy over another, but using them to guide exact stent positioning may be unrealistic without further advances.

## Modeling the Effect of Stenting Upon Ffr

In recent years, there has been a renewed interest in coronary physiology. FFR, a physiological parameter, is now considered the gold standard for assessing coronary artery lesion significance. FFR is measured during invasive angiography using a pressure-sensitive wire that is placed distal to the lesion. FFR is defined as the ratio of pressure distal to the lesion to the proximal pressure at maximal hyperemia. Attainment of maximal hyperemia is a requirement for accurate FFR assessment and is most commonly achieved with the infusion of intravenous adenosine, a vasodilatory drug. The resultant vasodilatation of the coronary microvasculature reduces the distal resistance, maximizing the flow rate of blood through the vessel. A threshold of 0.80 is applied to determine physiological lesion significance. If the FFR is <0.80, revascularization is recommended whereas if the FFR is >0.80, there is no indication for revascularization.

Using FFR to determine when PCI is required, is associated with improved clinical outcomes (De Bruyne et al., 2012). Computing coronary physiology, eliminating the need for invasive instrumentation, is an area of great interest. Several groups have developed methods to compute vFFR with varied success (Koo et al., 2011; Morris et al., 2013; Tu et al., 2014). Virtual stenting can be applied to these models, allowing a prediction of the likely improvement in physiology that can be achieved with stenting. Any desired width or length of stent can be modeled. For this, the details of the stent structure are not required, because we do not require details of flow disturbance at the stent/artery interface, reducing the complexity of the modeling.

### Inserting a “Virtual” Stent Using CT Imaging

Simulating the insertion of a virtual, cylindrical, stented segment into a modeled coronary vessel with recalculation of blood flow permits treatment planning. This allows operators to predict the physiological and anatomical response to treatment with different stent sizes in different locations, to plan the optimal solution before any treatment is delivered. This technique has recently been demonstrated with CTCA imaging (Kim et al., 2014). The investigators identified 44 patients with functionally significant lesions who underwent invasive angiography with FFR measurement. CTCA was performed prior to angiography and 3-D models of the coronary tree were reconstructed. Data on coronary flow and pressure were simulated using CFD. The pre-stent model was then marked for the location of stent used to treat the patient and a virtual stent was inserted to replicate the *in vivo* procedure. Subsequent FFR was computed following virtual stent implantation. The diagnostic accuracy to predict ischemia after PCI was 96%. The mean difference between vFFR and measured FFR after PCI was 0.024 (95% level of agreement -0.08 to 0.13). However, CTCA is still limited in its availability, most of these patients will still proceed to invasive angiography, and CTCA images can be limited by movement artifacts, poor heart rate control, and inaccuracy in calcific disease.

### Modeling Stenting Based Upon Invasive Angiography

**FIGURE 2:**
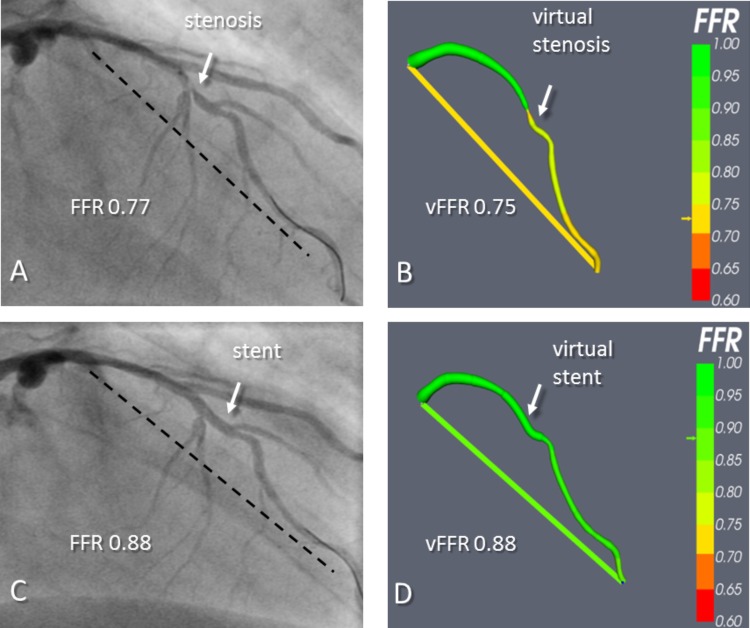
An example of virtual coronary intervention. Angiography revealed a severe mid vessel stenosis in the LAD (arrow). The mFFR between the proximal and distal points marked with the dashed line was 0.77. **(B)** The angiograms were used to model the vFFR using the VIRTUheart system, which was calculated to be 0.75 over the same segment. This is displayed as a straight yellow line connecting the same two points between which the vFFR was calculated, exactly matching the two spots marked by the dashed line in **(A)**. **(C)** After implantation of a 2.75 mm × 18 mm stent at the stenosis, the mFFR was 0.88 over the same segment. **(D)** VCI using the VIRTUheart system was then used to implant a “virtual” 2.75 mm × 18 mm stent, and the recalculated vFFR was 0.88, corresponding to a green color in the line connecting the two points. Reproduced from JACC: Cardiovascular imaging under creative commons license CC BY 4.0 (Gosling et al., 2018).

More recently, modeling the effect of stenting on FFR has been demonstrated by our group using invasive angiographic imaging (Gosling et al., 2018). This has the advantage of not using complementary imaging, so no co-registration of another modality to the angiogram is necessary, and the whole process is kept simple and readily interpretable by a conventional, angiogram-guided PCI. VCI was carried out in 54 patients (59 arteries) who underwent elective PCI. A 3-D reconstruction of the arterial geometry was created from the angiographic images. To validate the process, the size and position of stent(s) used *in vivo* was replicated using dedicated software (**Figure 2**). The authors demonstrated good accuracy in predicting the physiological response to stenting. Mean FFR post-PCI was 0.90 and mean vFFR post VCI was 0.92. The mean difference between vFFR after VCI and measured FFR after PCI was 0.01 ± 0.03. Importantly, the average computational time was just 2 min per case. Applying a VCI tool to invasive angiography will allow treatment planning to occur in the cardiac catheterization laboratory.

### What Is the Value of Virtual Treatment Planning?

A simple case with an isolated lesion may not require VCI. Interest will be concentrated on complex disease, such as arteries with serial lesions, diffuse disease, and bifurcations. Some outcome data suggests that patients who have a post-treatment FFR < 0.90 have increased risk of MACE at follow up (Pijls et al., 2002). The ability to predict the post treatment FFR for a particular stenting approach would potentially allow the operator to optimize the strategy prior to intervention, therefore improving both the post treatment FFR and clinical outcomes. Importantly, it can also allow the identification of patients unlikely to achieve significant improvement in FFR following PCI. This could help prevent unnecessary/futile procedures. VCI can also allow a more personalized assessment of a patient’s physiology. In the presence of serial stenoses, it is not possible to accurately measure the impact of one lesion upon the measured FFR across all the lesions due to the complex interplay of flow between them. Even FFR pullback is misleading. Therefore, deciding which lesion(s) to stent is challenging and often leads to unnecessary stenting (Pijls et al., 2000). Only by removing a stenosis (invasively by stenting it, which may not be necessary, or now “virtually” by computational modeling) is it possible to assess the effect of hyperemic flow across an individual stenosis among several.

## Immediate Next Steps

Although the potential for virtual stenting is clear, further work is required to validate treatment planning tools in patients with complex disease. The FFR-based tools may be more applicable than the models that employ complex WSS analysis. The technology is simpler and therefore the computational power required is significantly less. Rather than advising on exact positioning, they can provide a simpler recommendation of number and size of stent(s) which may be more translational. Moreover, the processing time is only a few minutes per case (Morris et al., 2017), with just slight improvements, “live” results are possible. This would be attractive to the interventionalist, who with the patient on the table, could get an immediate read out of optimal stent size and predicted response to their proposed strategy. This is important for clinical translation. In most cases, operators would not want to wait for overnight processing and then have to bring the patient back for their PCI procedure once the results are available.

### Future of PCI Treatment Planning Tools

Despite apparent success in the research domain, there are a number of challenges to be faced before PCI treatment planning tools can be incorporated into routine clinical practice. One of these is the computational power and time required to perform these analyses, in particular when FEM is used. Accuracy is key to success, but defining this is difficult, especially when there is no *in vivo* measure available to allow validation. All of the models are based upon a number of assumptions, which can affect their accuracy. The two key factors determining the accuracy of these models are the geometrical reconstruction and parameterization. Many of the models to date use reconstructions based upon CTCA, and although the accuracy of CTCA has improved in recent years, there are still a number of drawbacks including the translation of findings to those seen at invasive coronary angiography. The evolution of tools based upon the angiographic images may be a key advance. Accurate parameterization perhaps represents a more significant challenge. In many cases, much of the data required is readily available. However, predicting or calculating parameters that are not easily obtainable clinically, such as microvascular resistance, represents a major challenge and is perhaps the most significant factor hampering the accuracy of these models. Ultimately, demonstrating clinical success is vital and prospective randomized clinical trials will be required. There are also commercial considerations regarding accuracy and reliability of validation of such tools. The United States FDA is addressing this through a benchmarking initiative that aims to advance the application of CFD technology within the regulatory context and they have identified “developing computer modeling technologies” as a regulatory science priority (U.S Food and Drug Administration, 2017). Furthermore, the American Society of Mechanical Engineering (ASME) has produced standards for the verification and validation of computation fluid dynamics models (ASME, 2006, 2009). To allow widespread adoption of these tools, these, or similar approaches need to be extended to Europe. The final and perhaps most significant challenge is achieving acceptance within the clinical community. With modeling becoming a rapidly growing area, clinicians are increasingly encountering modeling-based technologies. The most recent example is the introduction of CT-FFR into national guidance (NICE, 2017). Yet, there is still some skepticism among many clinicians surrounding these technologies. Data from outcome studies will assist with this, but only with increased exposure over time, and a perseverance from the modeling community will wide spread acceptance be achieved.

## Conclusion

Computational modeling is routinely applied to assist with stent design, and there has been significant success in this area. More recently, the same technologies have been adapted to permit patient-specific virtual treatment planning. Complex models assessing WSS in bifurcation stenting can provide interesting insights into the relationships between stent design and stenting strategies on factors such as ISR. However, it is hard to see how they will impact clinical practice without significant simplification. Perhaps closer to clinical translation are models of FFR. These models permit prediction of post treatment FFR, a validated clinical measure, associated with different stenting strategies. Post PCI FFR is already established to be associated with clinical outcomes therefore the clinical benefit is clear. Moreover, the technology is simpler and therefore the processing time is substantially quicker. These models may not be too far from the clinical domain, but only time will tell.

## Author Contributions

RG and PM conceived the idea and wrote the first draft. All authors critically reviewed the paper and approved the final manuscript for submission.

## Conflict of Interest Statement

The authors declare that the research was conducted in the absence of any commercial or financial relationships that could be construed as a potential conflict of interest.

## ABBREVIATIONS

3-D: three dimensional
CAD: coronary artery disease
CFD: computational fluid dynamics
CTCA: computed tomography coronary angiography
CT-FFR: computed tomography fractional flow reserve
FDA: Food and Drug Administration
FEM: finite element modeling
FFR: fractional flow reserve
ISR: in-stent re-stenosis
IVUS: intravascular ultrasound
MACE: major adverse cardiac events
NPV: negative predictive value
OCT: optical coherence tomography
PCI: percutaneous coronary intervention
PPV: positive predictive value
VCI: virtual coronary intervention
vFFR: virtual fractional flow reserve
WSS: wall shear stress.
